# Biotensegrity or Fascintegrity?

**DOI:** 10.7759/cureus.4819

**Published:** 2019-06-03

**Authors:** Bruno Bordoni, Matthew A Varacallo, Bruno Morabito, Marta Simonelli

**Affiliations:** 1 Cardiology, Foundation Don Carlo Gnocchi, Milan, ITA; 2 Orthopaedic Surgery and Sports Medicine, University of Kentucky, Lexington, USA; 3 Osteopathy, School of Osteopathic Centre for Research and Studies, Milan, ITA; 4 Osteopathy, French-Italian School of Osteopathy, Pisa, ITA

**Keywords:** fascia, myofascial, skeletal muscle, shear stress, osteopathic, physiotherapy

## Abstract

The biotensegrity view of the living is a theoretical model and there is no mathematical study in vitro or in vivo that demonstrates its validity, taking into account the presence of liquids (blood, lymph, water), the tension produced by nerves and blood vessels, just as the displacement of the viscera and their resistances and contractions are not taken into consideration. The concept of cellular transduction is reviewed as it is the key to understanding if the passage of different mechanical information occurs only through solid structures, such as the cytoskeleton, or even liquid and viscous. The article focuses on reviewing the weaknesses of the biotensegrity model in the light of new scientific information, trying to coin another term that better reflects the dynamics of living: fascintegrity.

## Introduction and background

The designer R. Buckminster Fuller in the forties coined the term tensegrity, which derives from the contraction of the words tensional integrity, moving away from the concept of compressional continuity [1]. Tensegrity means an architectural structural principle where there is a breakdown of axial stresses (continuous tension with discontinuous compression); the structure is formed by compressed elements (usually bars or uprights) and by elements subjected to tension (cables), thus defining a new multi-polyhedral or vertical space. This structure creates its own balance, maintaining its shape [1]. In 1993, the term tensegrity is reported as a conceptual model towards the cell, highlighting how the organization of the living can be interpreted as a tensegrity structure. The living tissue, thanks to its organization with elements in continuous tension/detention, can correctly manage mechanical tensions and transmit them throughout the body (mechanotransduction) [1]. In this principle, we can find two biological structures: prestressed and geodesic [2]. The first structure is made up of elements that do not change their nature, that is, an element that manages the tensions and another element that produces them, respectively the microtubules and the actomyosin proteins. Geodesics can alternate their nature in producing tension or managing tension, as well as increasing or decreasing the elements involved that make up the biological form, for example, the complexity of a molecule [2]. According to the concept of tensegrity reported in the biological, the organization thus conceived of the structure allows restoring the original form, once the same structure has been deformed by the passage of mechanical tension, without damaging the components that constitute the tensegretive form [2]. The key concepts of the tensegretive model are elasticity, deformability, mechanical transmission and restoration of the form. Towards the end of the seventies, the term biotensegrity is coined, applying the tensegretive organization of the cell to the whole human body, as a fractal organization that repeats itself [2]. Biotensegrity is more complex, because it takes into consideration organs or tissues or systems, such as the musculoskeletal system or the fascial system, to explain the mechanics of the movement. Biotensegrity makes a subsequent step, that is, it not only allows the cell or the structure to survive and adapt, but allows it to move, explore, in schemes based on the concept of tensegrity [2]. Three other key points are highlighted: mobility, stability, and function. Biotensegrity is associated with the prestressed model, with soft elements (muscles, ligaments, tendons) and hard elements (bones, joints), such as the neck, the pelvis, the spine. The strength of this model is based on the presence of mechanotransduction at the cellular level. As Ingberg writes, tensegrity in biology is a theoretical view of how cells are able to adapt to mechanical deformations, stimulating biochemical responses, and allowing the cell to adapt and survive [1]. The biotensegretive view of living is a model and there is no theoretical, mathematical, in vitro or in vivo study that demonstrates its validity taking into account the presence of liquids (blood, lymph, water), the tension produced by nerves and blood vessels, just as we do not take into consideration the displacement of the viscera and their resistances and contractions. A cell without liquids does not move and cannot even adapt and survive. The logic behind the concept of cellular tensegrity and biotensegrity is the fact that all the structures that make up the cell up to the tissue are in connection (from the epidermis to the bone) [3]. To try to understand if this organization reflects the transmission of different mechanical tensions, it is necessary to review how the cell adapts to the passage of mechanical information (mechanotransduction), as it is the key to understand if the tensegretive structural organization reflects the reality of the living. The article has the focus of reviewing the weaknesses of the biotensegretive model considering new scientific information, trying to coin another term that better reflects the dynamics of the living.

## Review

Mechanotransduction

**Figure 1:**
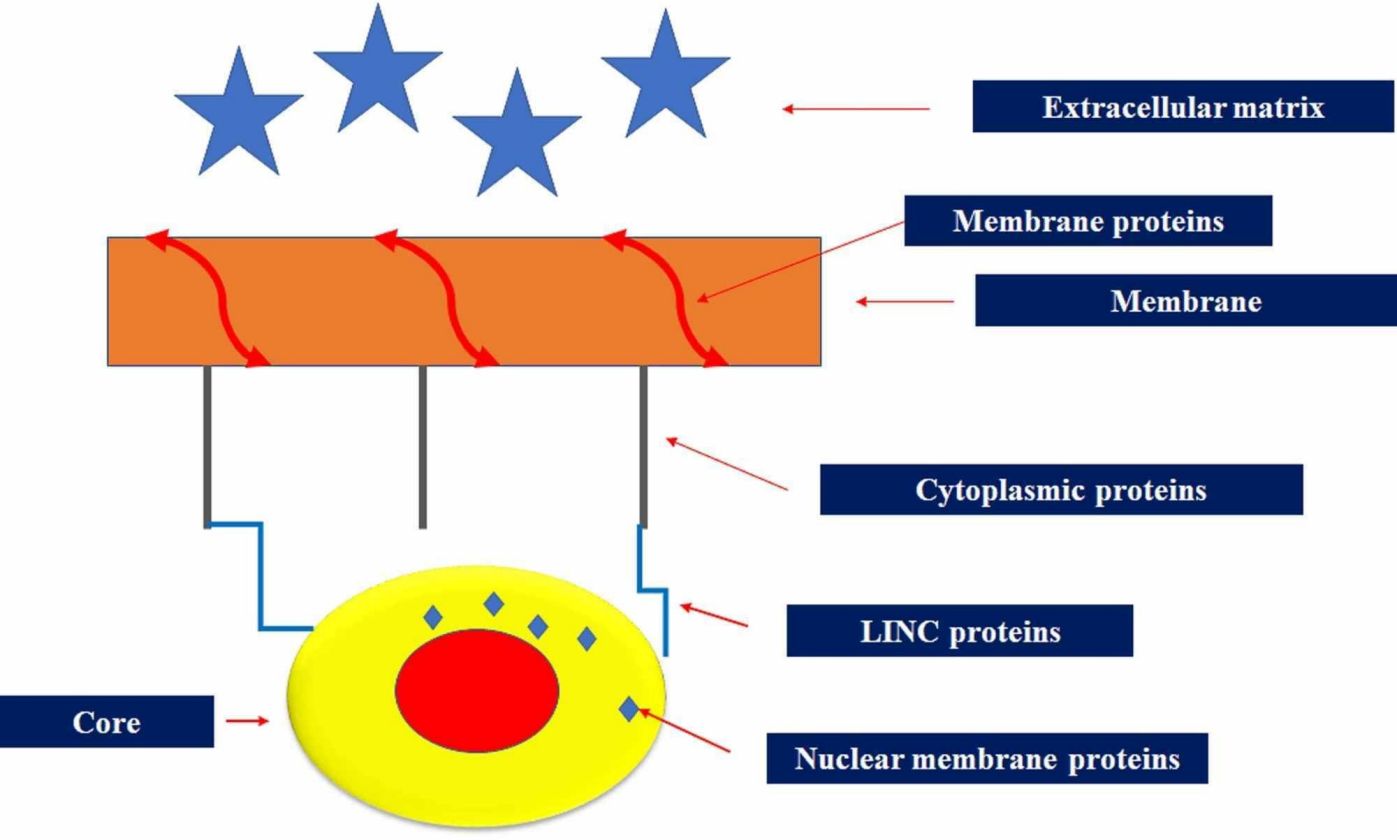
Transmission of the tension felt by the cell starting from the extracellular matrix The membrane proteins that interface with the matrix (focal adhesions proteins) transmit the mechanical tension felt towards the inside of the cell, thanks to the connection with other membrane proteins (integrins, dystrophins, dystroglycans, and others). The latter reports the tension to the cytoplasmic proteins (actomyosin, talin, paxillin, vinculin, and others), which will transmit the mechanical information to the nuclear membrane proteins (emerins and lamins), through the linker of nucleoskeleton and cytoskeleton or LINC proteins.

The cell is immersed in an environment of constant information, which stimulates and determines cellular behaviour, such as differentiation, migration and spreading: cellular survival depends on the ability to warn such information [4]. Mechanical information comes from the tension of the matrix extracellular, from stretching and shear stress of liquids [4]. The deformation of the cellular form in front of this information is the language that the cell uses with respect to its mechanometabolic environment. The cell dynamically senses deformation, restructuring the cytoskeleton and/or realigning its position. The realignment depends not only on the vector of mechanical force but also on the frequency of application of the force measured in hertz (Hz). The fibroblasts and the cells that make up the blood vessels, as well as the myocytes in the presence of a stretching force greater than 1Hz, preferentially undergo an alignment that is parallel to the vector of the applied force [4]. The cell realignment is also felt by the nucleus of the cell, which reconfigures its structure. This last event can happen thanks to the cytoskeletal proteins that interface with the extracellular matrix (elastin, fibronectin, laminins, and others), such as the focal adhesions proteins (FAKs), the membrane proteins (integrins, dystrophin, dystroglycan, and others) and cytoplasmic proteins (actomyosin, talin, paxillin, vinculin, and others) [5]. These structures carry the mechanical signal inside the cell and towards the nuclear membrane proteins, via the linker of nucleoskeleton and cytoskeleton or LINC proteins. The LINC proteins consist in particular of the nesprins of type 1 and 2; these are related to the cytoplasmic proteins and to the external nuclear membrane, where the nesprins connect with other proteins such as the emerins, the latter finally connected with the deeper proteins of the nuclear membrane such as the lamins, in contact with the chromaffin (Figure 1) [6-8].

**Figure 2:**
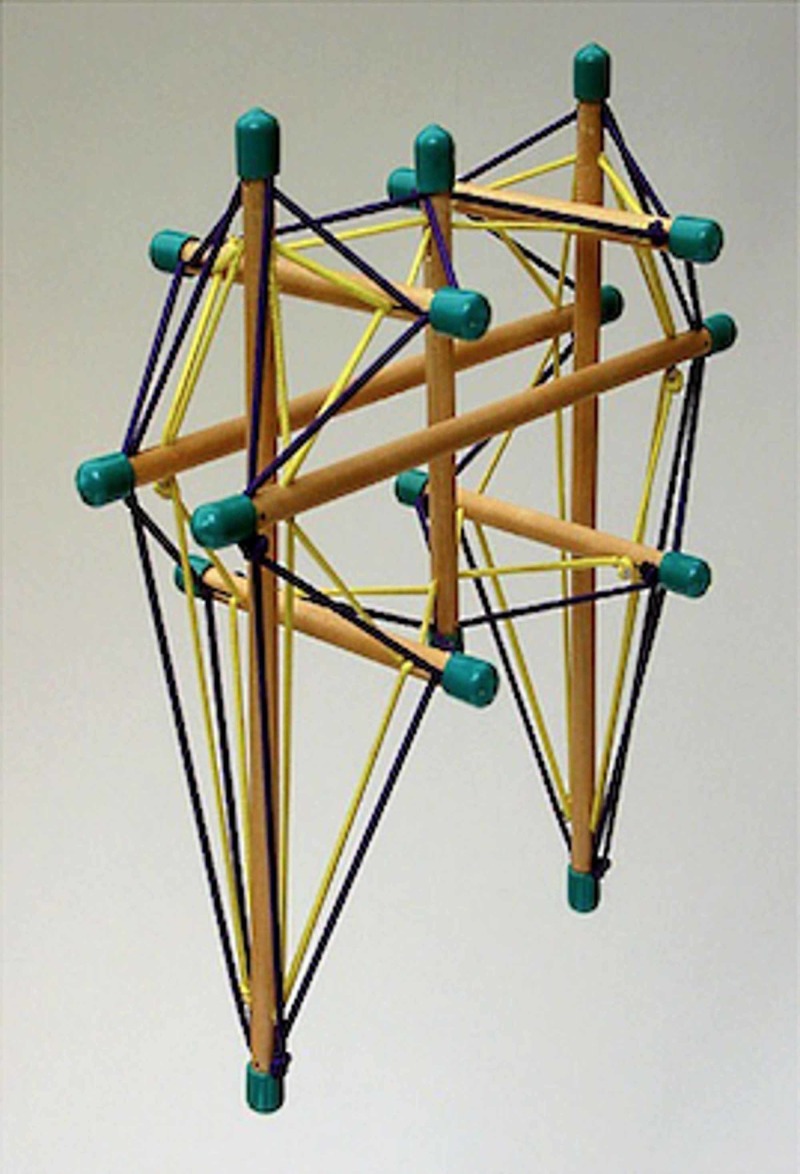
Mechanistic example of interpretation of biotensegrity It is a model that schematically represents the area of the pelvis and the lower limbs. The threads represent the structures in pre-stress such as the muscles, while the sticks represent the hard elements like bones.

With this model of mechanotransduction, the biotensegrity model is mirrored, where structures in pre-stress are proteins, while more rigid structures with the task of preserving integrity are the membranes (of the cell and the nucleus). The time required for the nucleus to sense mechanical information from the extracellular matrix and to effect a morphological rearrangement is about 10 minutes [4]. In reality, the first responses to mechanical information from the extracellular matrix are chemical, thanks to the ionic membrane channels sensitive to stretching, which cause different biochemical components to penetrate into the cytoplasm (adenosine triphosphate or ATP, calcium) in 10-20 seconds [4]. This chemical information binds to purinergic cytoplasmic ATP-dependent protein receptors, such as G-protein-coupled receptors (GPCRs), which activated receptors have the ability to realign cytoplasmic proteins and influence nuclear membrane tension, reshaping the core [4]. Other biochemical factors take over this process to improve nuclear remodelling, such as transforming growth factor-beta (TGF-β); the latter is synthesized by the tension acquired by the membrane through the extracellular matrix. The TGF-β activated in the membrane and in contact with the cytoplasm and the extracellular matrix, binds to the small mother against decapentaplegic proteins (Smad type 1,2 and 3) and to Ras-homologous (Rho) protein family. TGF-β/Smad and TGF-β/Rho signalling are complexes able to influence gene expression [9]. The cellular organization and the relationship with the structures of the extracellular matrix according to the vision of biotensegrity, multiplying the cell in fractal mode, would also be found in the complexity of the skeletal-muscular segments (Figure 2).

Liquids and mechanotransduction

**Figure 3:**
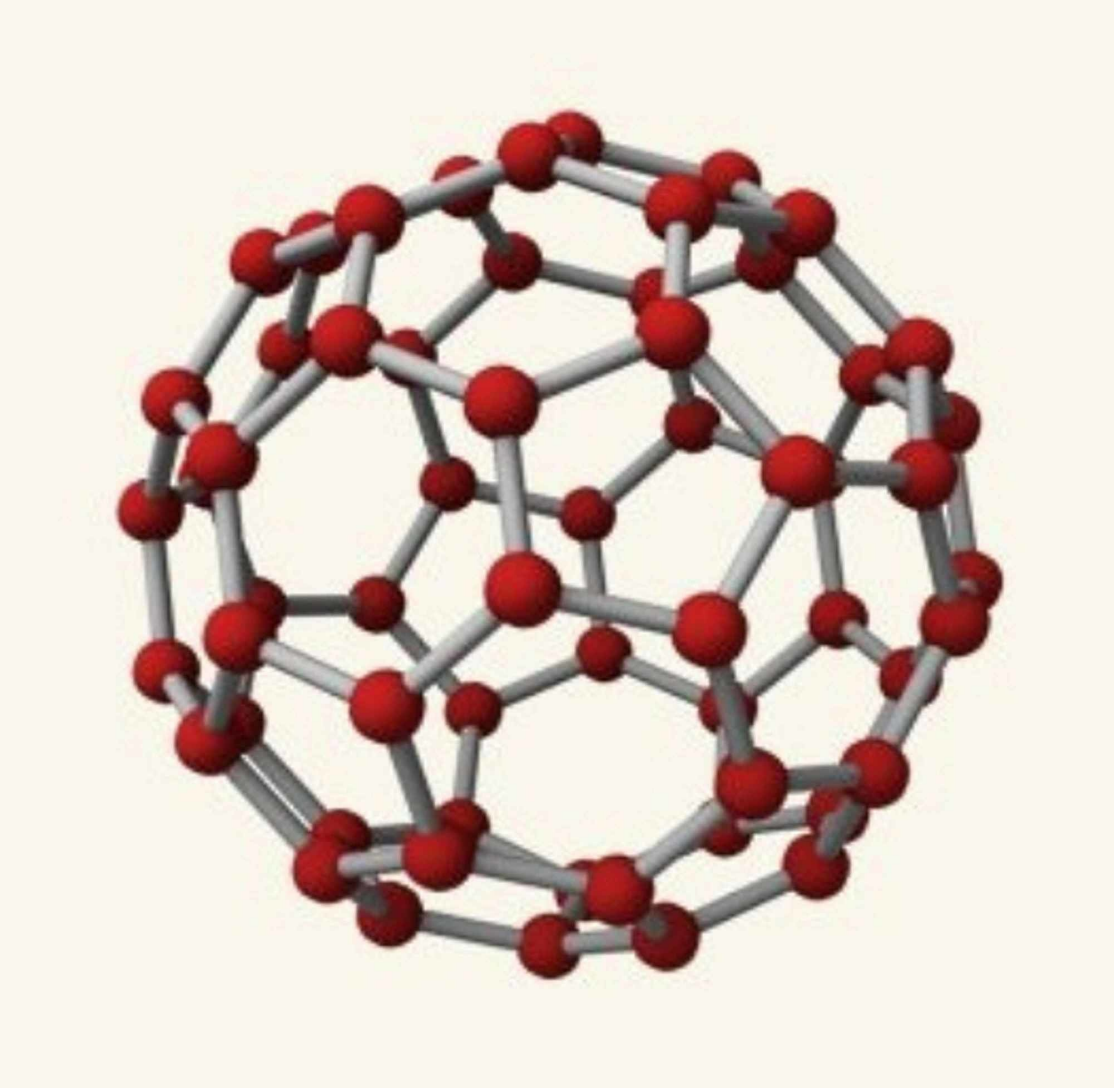
A molecule model to represent the biotensegrity idea of a geodesic type

Another model to explain the capacity of cellular adaptation is through the presence of liquids inside the cell or outside the cell. A cell is able to undergo and adapt both with a mechanical stimulus coming from the stretching/shortening of the membrane and coming from a liquid stimulus or shear stress [4]. The cell subjected to constant fluid passages tends to orient its structure in the direction of the liquid, such as endothelial cells with the passage of blood or myocytes influenced by the pressure of the liquids of the extracellular matrix [4,10]. The current biotensegretive model does not take into consideration of the presence of liquids (blood, lymph, cytoplasm, extracellular matrix) and neither the ability of liquids to influence the shape and health of cells and tissues. The cell without the liquids does not move and cannot survive. The biotensegretive model is based on mechanical patterns of the structure (for example a molecule) but in the absence of liquids (Figure 3) [11].

Interstitial Fluids

The cell does not house the liquid but is the host of liquids. Liquids surround the cell (extracellular matrix), liquids are found inside the membrane and inside the cell (cytoplasm). The interstitial fluids (IFs) that make up the extracellular matrix move driven by pressure gradients at a speed of about 0.1-4.0 micrometres per second (µm/s); the different speed will depend on the hydrostatic and osmotic pressure differences between the blood and Ifs [12]. This movement or shear stress creates a deformation of the membrane, capable of stimulating cell morphology. IFs can create tangential, drag or wave forces influencing the composition of the same matrix or the alignment of the collagen, and the behaviour/remodelling of the cell [12]. In the presence of shear stress the alpha-actinin and in particular the membrane filamin transmit the force felt to the cytoskeletal actin, which refers the information to the FAKs; the latter activate biochemical pathways that will allow cytoplasmic proteins to align with the direction of the liquid stimulus, such as Rho and Ras-related GTP-binding protein (Rac) [4]. If the cell is parallel to the flow of IFs the mechanical deformation will be slight, while if the cell is positioned perpendicular or slightly oblique with respect to the flow direction of interstitial fluids, the mechanical stimulation that will result will be greater. In the latter case, the cell will be strongly influenced by liquids [12]. If liquids find an extracellular matrix environment with little space and a lot of solid material (fibroblasts, collagen, proteoglycans, glycosaminoglycans), there will be a low compliant mechanometabolic environment, guiding most solid structures, for example fibroblasts, towards an even more rigid area (durotaxis or migration to an area of greater stiffness) [4,13]. The flow of liquids outside the cell is fundamental for a balanced tension/pressure of the extracellular matrix and to allow the cell to correctly perceive not only the flow of liquids but also to obtain correct tensional information from solid structures from the matrix [12]. Liquids are not structures attributable to the concept of pre-stress, but yet, we find fluids also inside the cell membrane.

Fluid-Mosaic Model

According to the membrane model devised in 1972 but always up to date, known as the fluid-mosaic model, we can imagine the cell membrane as a double layer of phospholipids, with globular and fibrous proteins, channel-forming proteins (ionic or biochemical) and carbohydrate chains [14]. The membrane is in direct contact with the solid and liquid structures of the extracellular matrix and with the inside of the cell itself. The structures that make up the membrane (transmembrane or cis-membrane) can move from their original location, thus changing their function, their ability to interact with the tensions felt or change their ability to produce tension [14]. This shift is determined above all from the pressures that reach the membrane from the extracellular matrix, from the cytoplasm and from the plasmatic liquids of the same membrane [14]. This determines that the biological environment where the cells of our body live is, first of all, a liquid environment [15-16]. The cell membrane transports the tension felt uneven, and not all the membrane responds in unison to the same tension. A membrane deformation can activate biochemical responses only locally or activate distant replies with respect to where the primary mechanical deformation occurred [17]. The concept of biotensegrity must necessarily be revised, since, although there is a solid continuity of the different structures of the living, liquids govern the form and function of these structures; the same membrane does not mechanically and schematically transmit the tension felt according to the tensegretive principle. Furthermore, the membrane structures themselves shift, changing their nature from pre-stress to more rigid structures, mixing the concept of biotensegrity. The aggregates of cells move like a liquid in the interstitial space and in the interstitial fluid and this vision has allowed us to understand the behaviour of the tissues [18].

Shear Stress

The shear stress of the interstitial fluid is extraordinarily important for the shape and function of the cells, allowing to create the shape and function of tissues, such as bone, epithelial and muscle [19-21]. The shear stress generated by interstitial fluids during muscle contraction is a more powerful adaptation stimulus than the mechanical stimulus that shortens and lengthens the muscle fibres [21]. All muscles perceive shear stress thanks to the extracellular matrix fluids, with a response of substances usually involved in the mechanism of hypertrophy in a higher percentage (about twice), compared to the contraction and relaxation of contractile fibers only: insulin-like growth factor -1 (IGF-1), mechano growth factor (MGF), vascular endothelial growth factor (VEGF), and nitric oxide (NO) [21]. Without the presence of liquids and relative shear stress, tissue adaptation does not occur.

Cytoplasm

**Figure 4:**
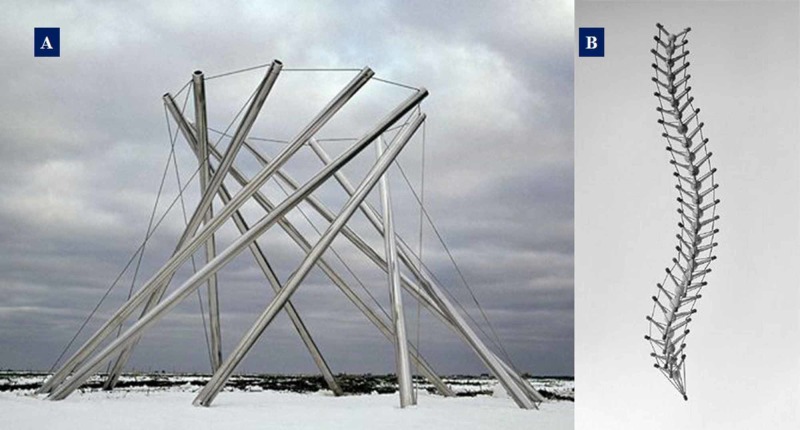
Image A: A work by the architect Kenneth Snelson and a student of Dr Fuller (Columbus Museum of Art, Columbus, OH), whose work represents the concept of tensegrity in architecture; image B: a model of the vertebral column with the concept of biotensegrity

The inside of the cell is rich in the cytoplasm. What allows the mechanical signals coming from the membrane deformation (stretching or shear stress), and the biochemical signals to reach the nucleus quickly, is the presence of the cytoplasm, a viscous substance. The cytoplasm is not attributable to a pre-stress or rigid structure but yet it plays an extraordinary role in the mechanotransduction and in the passage of tensional information [4]. Thanks to the cytoplasm, the cell can be considered as a liquid motor [22]. The actomyosin complex that constitutes the scaffolding of each cell and capable of moving, moves the cytoplasm into the area of the cell where there is less stiffness. Myosin draws actin towards the inside of the cell so that on the one hand the cell is not damaged by mechanical deformation and on the other, it is useful for directing the information recorded by actin towards myosin. The contraction and the tension produced are able to create oscillating cytoplasmic waves, which will create biochemical information for the remodelling of the cell: shear stress inside the cell [22-23]. These cytoplasmic waves will determine the shape and function of the cell and will influence the mode of contraction of the actomyosin network [22-23]. It is the cytoplasmic fluid that determines how the cell moves and contracts. The cytoplasm has no rigid or pre-stress components and cannot be associated with the biotensegretive or tensegretive model. The cell and the fractal organization that leads to the tissue of the living should be reviewed and conceived not as the biotensegretive structures. Probably, the mistake was to try to understand the behaviour of the cell and body systems through an architectural model (schematic and without liquids; Figure 4).

When creating a model, it must be remembered that as a model it must change and must be malleable, depending on the new information emerging from the research, so that the understanding of what you want to represent is as close as possible to reality. We quote a sentence from Prof. Nicolson: “It is rare that scientific models are not modified from their original forms to reflect new observations or data that were not anticipated when the models were proposed [14].”

Fascintegrity

The term "fascintegrity" is the union of the words fascia and integrity. In recent works we have given a new definition of fascia: “The fascia is any tissue that contains features capable of responding to mechanical stimuli. The fascial continuum is the result of the evolution of the perfect synergy among different tissues, liquids and solids, capable of supporting, dividing, penetrating, feeding and connecting all the districts of the body: epidermis, dermis, fat, blood, lymph, blood and lymphatic vessels, tissue covering the nervous filaments (endoneurium, perineurium, epineurium), voluntary striated muscle fibers and the tissue covering and permeating it (epimysium, perimysium, endomysium), ligaments, tendons, aponeurosis, cartilage, bones, meninges, involuntary striated musculature, and involuntary smooth muscle (all viscera derived from the mesoderm), tongue. The continuum constantly transmits and receives mechano-metabolic information that can influence the shape and function of the entire body. These afferent/efferent impulses come from the fascia and the tissues that are not considered as part of the fascia in a bi-univocal mode [24,25].” The fascia has solid and liquid components (blood and lymph), which fascia conditions the shape and function of the living (from the cell to the tissue), puts the whole body in communication, and influences the movement. The fascia is not divided into layers, as each cell of different tissues is in communication with other cells of other tissues and able to condition each other not only from the mechanical point of view but also from the biochemical and liquid point of view [3,11,26-27]. To understand functional anatomy and the behaviour of the living one should not take an example from an architectural scaffolding, but from a living example, like the fascia. The fascia contains solid and liquid elements and the presence of liquids is the missing element to the biotensegretive model. The pressure and direction of blood and lymph affect the muscles and joints, as the muscles and joints are the results of an asynchronous fascial fractal summation of what the liquids impose [3,11]. Liquids such as blood, lymph, extracellular matrix liquids and cytoplasm are not pre-stress elements or rigid elements. The fascia contains and is also constituted by the viscera, which tensions and movements are not considered in the biotensegretive model. The viscera condition posture and movement with their muscular contraction through involuntary neural patterns; the viscera feel the tension that surrounds them (blood, lymph, viscera, and skeletal muscles), changing their own tension [28-34]. Fascintegrity is the word that should be used to construct a new model to understand the adaptation behaviour of cells and tissues, as well as musculoskeletal movement. The integrity is given by the fractal and entropic organization that from the cell reaches the epidermis, which structures, solid and liquid, are fascia.

## Conclusions

The article has revised the concepts of tensegrity and biotensegrity, highlighting the fact that these models date back to the last century. Each model assumes and this assumption changes over time, thanks to scientific innovation; changing the construction of the model allows us to reflect the scientific innovations and allows us to project new concepts and thoughts forward, laying the foundations for constant improvement. We proposed a new term to stimulate further research into the understanding of cellular behaviour and body movement: fascintegrity. The term fascintegrity is the union of the words fascia and integrity. The human body is living integrity allowed by the fascial organization, including liquids and viscera.
